# Comparison of N···I and N···O Halogen Bonds in Organoiodine Cocrystals of Heterocyclic Aromatic Diazine Mono-*N*-oxides

**DOI:** 10.1021/acs.cgd.3c01344

**Published:** 2024-03-05

**Authors:** Clifford W. Padgett, Riley Dean, Audrey Cobb, Aubree Miller, Andrew Goetz, Sam Bailey, Kyle Hillis, Colin McMillen, Sydney Toney, Gary L. Guillet, Will Lynch, William T. Pennington

**Affiliations:** † Department of Biochemistry, Chemistry and Physics, Georgia Southern University, Savannah, Georgia 31419, United States; ‡ Department of Chemistry, Clemson University, Clemson, South Carolina 29634-0973, United States

## Abstract

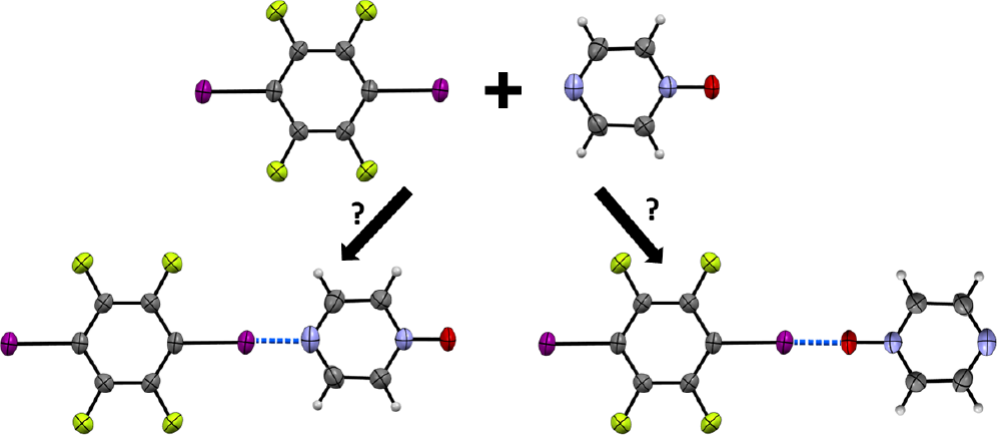

A series of cocrystals of halogen bond donors 1,4-diiodotetrafluorobenzene (*p*-F_4_DIB) and tetraiodoethylene (TIE) with five aromatic heterocyclic diazine mono-*N*-oxides based on pyrazine, tetramethylpyrazine, quinoxaline, phenazine, and pyrimidine as halogen bonding acceptors were studied. Structural analysis of the mono-*N*-oxides allows comparison of the competitive occurrence of N···I vs O···I interactions and the relative strength and directionality of these two types of interactions. Of the aromatic heterocyclic diazine mono-*N*-oxide organoiodine cocrystals examined, six exhibited 1:1 stoichiometry, forming chains that utilized both N···I and O···I interactions. Two cocrystals presented 1:1 stoichiometry with exclusive O···I interactions. Two cocrystals displayed a 2:1 stoichiometry—one characterized solely by O···I interactions and the other solely by N···I interactions. We have also compared these interactions to those present in the corresponding diazines, some of which we report here and some which have been previously reported. In addition, a computational analysis using density functional theory (M062X/def2-SVPD) was performed on these two systems and has been compared to the experimental results. The calculated complex formation energies were, on average, 4.7 kJ/mol lower for the I···O halogen bonding interaction as compared to the corresponding N···I interaction. The average I···O interaction distances were calculated to be 0.15 Å shorter than the corresponding I···N interactions.

## Abstract 2

Competitive nitrogen−iodine and oxygen−iodine halogen bonding studies using N-hetero diazine mono-*N*-oxides: crystallographic and computational studies.

## 1 Introduction

Halogen bonding (XB) has received much attention in the field of crystal engineering, due to the strength and directionality of these interactions.^1−6^ Halogen bonds are a type of Lewis acid/base interaction that involve the donation of a lone-pair of electrons from a donor atom to the σ* orbital, σ-hole, of an acceptor atom (in this case an iodine atom).^7,8^ These halogen bonding interactions are often referred to using a nomenclature similar to that of hydrogen bonding, where the electron pair acceptor is the halogen bond donor (XBD), and the electron pair donor is the halogen bond acceptor (XBA). Halogen bonding interactions are reasonably strong,^9^ highly directional,^10−12^ and selective,^13−17^ making them suitable for geometry-based crystal design.^18,19^

Of the halogens, the highly polarizable diiodine molecule typically forms stronger interactions and is less prone to oxidation of the electron pair donor molecule, which makes diiodine, and polyiodides in general, useful choices for halogen-bonding-directed crystal engineering.^20−25^ However, diiodine is still a potent oxidizer and, thus, can be limited in its potential utility as a supramolecular building block. These limitations can be addressed by utilizing other forms of iodine that are less oxidizing, such as organoiodines.^26−31^ By inserting an organic ‘spacer’ between the two iodine atoms, the functionality of a polarized iodine atom can be retained while it exhibits less oxidation strength. The organic spacers also offer a means for influencing the Lewis acidity of the electron pair acceptors in these compounds by adjusting the electron density around the iodine atom.^32^ This can be achieved by selecting molecules that have different electron-withdrawing substituents present in addition to the iodine atoms or that have multiple iodine atoms located in different isomeric orientations. Organoiodines containing multiple iodine atoms additionally assist in the formation of multidirectional networks or complex discrete units via halogen bonding.^33−38^

Significant research efforts have been focused on the halogen bonding interaction between nitrogen-based Lewis bases and carbon-bonded halogens (mostly iodine). Other Lewis bases (halogen bond acceptors) like oxygen and sulfur have been comparatively less explored, though they offer promising versatility.^39−44^ Recent efforts in this area have identified *N*-oxides as effective halogen bond acceptors for both I_2_ and some organoiodine compounds.^45−52^

**Scheme 1:**
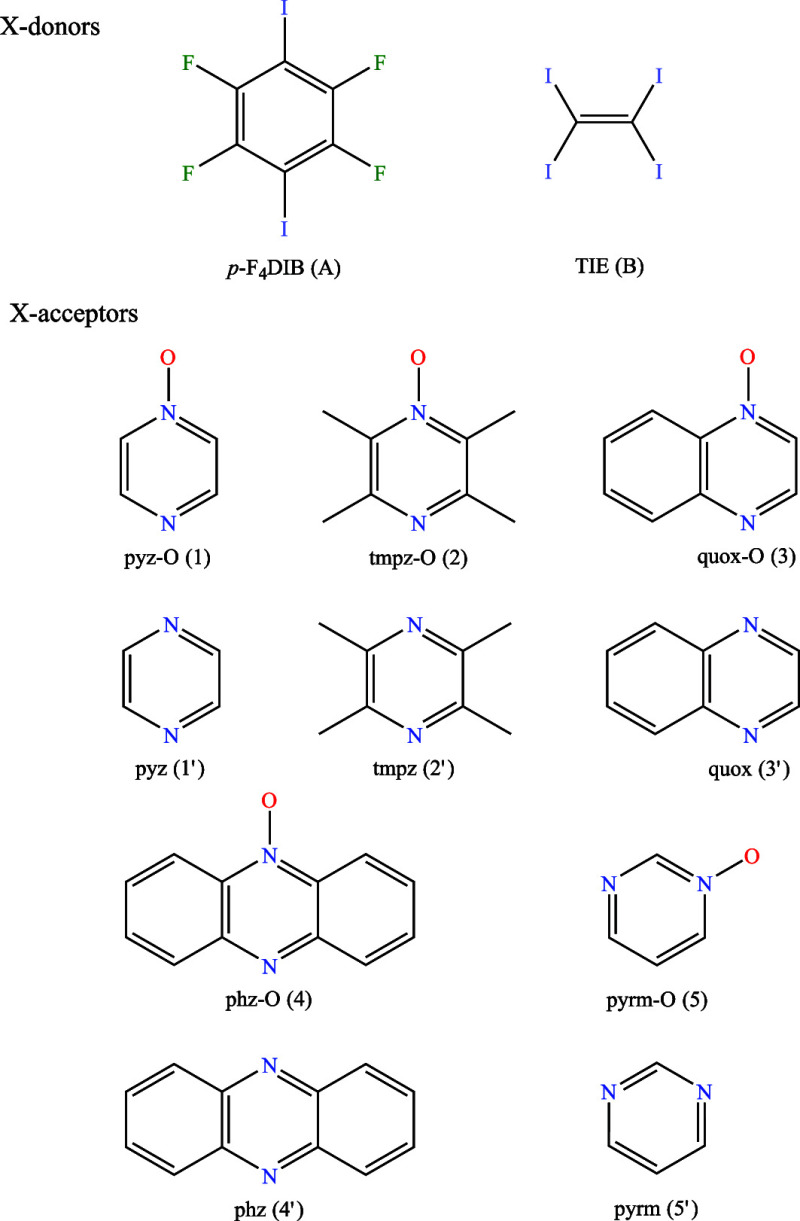
Scope of Halogen Bond Donors (**A**, **B**) and Acceptors (**1**, **2**, **3**, **4**, **5**) in the Present Study

Of particular interest to us is to perform experimental and computational structural comparisons of X···O vs X···N interactions in aromatic heterocyclic diazine mono-*N*-oxides, where both nitrogen and oxygen atoms are available as halogen bond acceptors. These compounds allow for competitive nitrogen–iodine and oxygen–iodine halogen bonding and for the characterization of the strength and directionality of the resulting oxygen–halogen bonding interactions. Additionally, the study provides an opportunity to compare these interactions in the organoiodine-mono-*N*-oxide cocrystals to those in the corresponding organoiodine-diazine cocrystals, some of which are already reported in the structural literature.^53−57^ In the instances where crystallographic data are not available for this latter class of cocrystals, we also sought to prepare the corresponding cocrystal and refine its structure. Herein, we focus on the cocrystals formed by 1,4-diiodotetrafluorobenzene (*p*-F_4_DIB, **A**) and tetraiodoethylene (TIE, **B**) with the mono-*N*-oxides of pyrazine (pyz-O, **1**), tetramethylpyrazine (tmpz-O, **2**), quinoxaline (quox-O, **3**), phenazine (phz-O, **4**), and pyrimidine (pyrm-O, **5**) (Scheme 1).

## 2 Experimental Section

### 2.1 Reagents and General Procedures

The following reagents were commercially obtained and used without further purification: 1,4-diiodotetrafluorobenzene (Synquest Laboratories, 97%), tetraiodoethylene (Santa Cruz Biotechnologies, 98%), tetramethylpyrazine (Sigma-Aldrich, 98%), quinoxaline (Sigma-Aldrich, 98%), pyrimidine (Acros Organics, 97%), pyrazine-*N*-oxide (Sigma-Aldrich, 97%), pyrimidine-*N*-oxide (Santa Cruz Biotechnologies, 97%), and phenazine *N*-oxide (Sigma-Aldrich, 98%).

### 2.2 Synthesis of Mono-*N*-oxides

Mono-*N*-oxides that were not obtained commercially were prepared as follows.

#### 2.2.1 Synthesis of 2,3,5,6-Tetramethylpyrazine-N-oxide

2,3,5,6-Tetramethylpyrazine (1.0097 g, 7.4139 mmol) was charged into a reaction flask with 15 mL of dichloromethane and cooled to 0 °C. A 30 mL solution made from 70 to 75% meta-chloroperoxybenzoic acid (mCPBA) (1.5048 g, ∼6.5 mmol) was added dropwise to the cooled reaction, and the reaction was allowed to warm to room temperature. The reaction was stirred for 48 h. The solvent was removed under reduced pressure, and then the reaction residue was loaded onto a silica gel column using a dry plug. The column was run with 7:1 ethyl acetate/methanol as eluent with the desired product as the third component to elute following the mCPBA and tetramethylpyrazine fractions. The solvent was removed from the 2,3,5,6-tetramethylpyrazine-*N-*oxide fraction yielding 0.6638 g of pure white solid in 52% yield. ^1^H NMR (300 MHz, CDCl_3_, 300 K): δ 2.46 (s, 3H), 2.51 (s, 3H).

#### 2.2.2 Synthesis of Quinoxaline-N-oxide

Quinoxaline (1.0122 g, 8.156 mmol) was charged into a reaction flask with 35 mL of chloroform and cooled to 0 °C. A 25 mL solution made from 70 to 75% mCPBA (1.8806 g, ∼8.2 mmol) in chloroform was added dropwise to the cooled reaction, and the reaction was allowed to warm to room temperature. The reaction was stirred for 48 h. The solvent was removed under reduced pressure and then the reaction residue was loaded onto a silica gel column using a dry plug. The column was run with ethyl acetate as eluent with the desired product as the second component to elute following the mCPBA fraction. The solvent was removed from the quinoxaline-*N*-oxide fraction yielding 0.7618 g of pure white solid in 66.6% yield. ^1^H NMR (300 MHz, CDCl_3_, 300 K): δ 7.73–7.86 (m, 2H), 8.13 (d, 1H), 8.35 (d, 1H), 8.58 (d, 1H), 8.67 (d, 1H).

### 2.3 Synthesis of Cocrystals

#### 2.3.1 Synthesis of 2(pyz-O)·p-F_4_DIB **(1A)**

Pyrazine-*N*-oxide (0.048 g, 0.50 mmol) and 1,4-diiodotetrafluorobenzene (0.061 g, 0.15 mmol) were combined in a 100 mL beaker and dissolved in approximately 50 mL of dichloromethane, after which the beaker was covered with parafilm. The solvent was allowed to evaporate at room temperature for several days. A colorless irregularly shaped crystal was selected for X-ray analysis.

#### 2.3.2 Synthesis of tmpz-O·p-F_4_DIB (**2A**)

Tetramethylpyrazine-*N*-oxide (0.022 g, 0.16 mmol) and 1,4-diiodotetrafluorobenzene (0.066 g, 0.16 mmol) were combined in a 100 mL beaker and dissolved in approximately 50 mL of dichloromethane, after which the beaker was covered with parafilm. The solvent was allowed to evaporate at room temperature over a period of several days. A colorless plate-like crystal was selected for X-ray analysis.

#### 2.3.3 quox-O·p-F_4_DIB (**3A**)

Quinoxaline-*N*-oxide (0.020 g, 0.15 mmol) and 1,4-diiodotetrafluorobenzene (0.070 g, 0.17 mmol) were combined in a 100 mL beaker and dissolved in approximately 50 mL of dichloromethane, after which the beaker was covered with parafilm. The solvent was allowed to evaporate at room temperature over a period of several days. A colorless plate-like crystal was selected for X-ray analysis.

#### 2.3.4 Synthesis of 2(phz-O)·p-F_4_DIB (**4A**)

Phenazine-*N*-oxide (0.10 g, 0.51 mmol) and 1,4-diiodotetrafluorobenzene (0.10 g, 0.25 mmol) were combined in a 100 mL beaker and dissolved in approximately 50 mL of dichloromethane, after which the beaker was covered with parafilm. The solvent was allowed to evaporate at room temperature over a period of several days. A slightly orangish needle-like crystal was selected for X-ray analysis.

#### 2.3.5 Synthesis of pyrm-O·p-F_4_DIB (**5A**)

Pyrimidine-*N*-oxide (0.024 g, 0.25 mmol) and 1,4-diiodotetrafluorobenzene (0.10 g, 0.25 mmol) were combined in a 20 mL vial and dissolved in 10 mL of dichloromethane. The solvent was allowed to evaporate at room temperature over a period of 2 days. A colorless block-like crystal was selected for X-ray analysis.

#### 2.3.6 Synthesis of Pyz-O·TIE (**1B**)

Pyrazine-*N*-oxide (0.10 g, 1.0 mmol) and tetraiodoethylene (0.10 g, 0.19 mmol) were combined in a 100 mL beaker and dissolved in approximately 50 mL of dichloromethane, after which the beaker was covered with parafilm. The solvent was allowed to evaporate at room temperature over a period of several days. A colorless irregular-shaped crystal was selected for X-ray analysis.

#### 2.3.7 Synthesis of 2(tmpz-O)·TIE (**2B**)

Tetramethylpyrazine-*N*-oxide (0.10 g, 0.73 mmol) and tetraiodoethylene (0.10 g, 0.19 mmol) were combined in a 100 mL beaker and dissolved in approximately 50 mL of dichloromethane, after which the beaker was covered with parafilm. The solvent was allowed to evaporate at room temperature over a period of several days. A colorless block-like crystal was selected for X-ray analysis.

#### 2.3.8 Synthesis of Quox-O·TIE (**3B**)

Quinoxaline-*N*-oxide (0.10 g, 0.67 mmol) and tetraiodoethylene (0.10 g, 0.19 mmol) were combined in a 100 mL beaker and dissolved in approximately 50 mL of dichloromethane, after which the beaker was covered with parafilm. The solvent was allowed to evaporate at room temperature over a period of several days. A colorless block-like crystal was selected for X-ray analysis.

#### 2.3.9 Synthesis of Phz-O·TIE (**4B**)

Phenazine-*N*-oxide (0.10 g, 0.51 mmol) and tetraiodoethylene (0.10 g, 0.18 mmol) were combined in a 100 mL beaker and dissolved in approximately 50 mL of dichloromethane, after which the beaker was covered with parafilm. The solvent was allowed to evaporate at room temperature over a period of several days. A clear irregular-shaped crystal was selected for X-ray analysis.

#### 2.3.10 Synthesis of Pyrm-O·TIE (**5B**)

Pyrimidine-*N*-oxide (0.024 g, 0.25 mmol) and tetraiodoethylene (0.13 g, 0.24 mmol) were combined in a 20 mL vial and dissolved in 15 mL of methanol with gentle heating. The solvent was allowed to evaporate at room temperature over a period of 5 days. A colorless columnar crystal was selected for X-ray analysis.

#### 2.3.11 Synthesis of pyrm·p-F_*4*_*DIB* (**5′A**)

1,4-Diiodotetrafluorobenzene (0.04 g, 0.1 mmol) was dissolved in 0.8 mL of neat pyrimidine in a 20 mL vial. The solvent was allowed to evaporate at room temperature over a period of 2 weeks. A colorless block-like crystal was selected for X-ray analysis.

#### 2.3.12 *Synthesis of Tmpz·TIE* (**2′B**)

Tetramethylpyrazine (0.02 g, 0.2 mmol) and tetraiodoethylene (0.08 g, 0.2 mmol) were combined in a 20 mL vial and dissolved in 15 mL of ethanol with gentle heating. The solvent was allowed to evaporate at room temperature over a period of 5 days. A colorless plate-like crystal was selected for X-ray analysis.

#### 2.3.13 *Synthesis of Pyrm·TIE* (**5′B**)

Tetraiodoethylene (0.04 g, 0.08 mmol) was dissolved in 0.8 mL of neat pyrimidine in a 20 mL vial. The solvent was allowed to evaporate at room temperature over a period of 2 weeks. A colorless block-like crystal was selected for X-ray analysis.

### 2.4 X-Ray Crystallography

Single-crystal X-ray diffraction data were obtained using Mo Kα radiation (λ = 0.71073 Å) with a Rigaku XtaLAB mini diffractometer (sealed Mo tube, Mercury 3 CCD, 170 K) or Bruker D8 Venture diffractometer (Mo microfocus tube, Photon II detector, 100 K) via rotations of φ and ω. Data were collected, processed, and corrected for absorption using CrysAlis Pro and Apex 3 (SAINT, SADABS) software.^58,59^ Structure solution and space group determination was performed using intrinsic phasing SHELXT,^60^ with subsequent refinement by full-matrix least-squares techniques on *F*^2^ using SHELXL^61^ and Olex2.^62^ All non-hydrogen atoms were refined anisotropically, and hydrogen atoms were refined in calculated positions using riding models with *U*_eq_(H) = 1.200*U*_eq_(C). Disordered atoms subject to symmetry constraints in the structures of **2A**, **5A**, and **1B** were refined in half-occupancy. The occupancies of the disordered oxygen atoms in **2B** were freely refined with a unity sum. The structures of **3B** and **5B** were refined as inversion twins with absolute structure parameters (Flack) of 0.48(7) and 0.18(9), respectively. Details of the structure refinements are summarized in Tables 1–2,3. Crystal packing diagrams are provided in the Supporting Information, Figures S1–S3. Crystallographic data may be obtained in CIF form from the Cambridge Crystallographic Data Centre via www.ccdc.cam.ac.uk/data_request/cif (or from the CCDC, 12 Union Road, Cambridge CB2 1EZ, UK; Fax: + 44 1223 336 033; E-mail: data_request@ccdc.cam.ac.uk) upon quoting deposition numbers 2297606–2297618.

**Table 1:** Crystallographic Data for Cocrystals **1A**–**5A**

	**1A2(pyz-O)·***p***-F**_**4**_**DIB**	**2Atmpz-O·***p***-F**_**4**_**DIB**	**3Aquox-O·***p***-F**_**4**_**DIB**	**4A2(phz-O)·***p***-F**_**4**_**DIB**	**5Apyrm-O·***p***-F**_**4**_DIB
empirical formula	C_14_H_8_F_4_I_2_N_4_O_2_	C_14_H_12_F_4_I_2_N_2_O	C_14_H_6_F_4_I_2_N_2_O	C_30_H_16_F_4_I_2_N_4_O_2_	C_10_H_4_F_4_I_2_N_2_O
*M*_*r*_ (g/mol)	594.04	554.06	548.01	794.27	497.95
crystal system	monoclinic	monoclinic	monoclinic	monoclinic	monoclinic
space group, *Z*	*C*2/*m*, 2	*P*2_1_/*n*, 2	*P*2_1_/*c*, 4	*P*2_1_/*c*, 2	*C*2/*c*, 4
temperature (K)	170(2)	170(2)	170(2)	170(2)	100(2)
*a* (Å)	8.7665(5)	14.158(5)	12.0858(17)	4.1448(3)	7.6566(3)
*b* (Å)	7.3072(5)	4.4441(10)	4.4328(5)	25.4812(19)	9.1097(3)
*c* (Å)	13.9543(10)	14.914(4)	29.258(3)	12.8493(9)	18.3856(8)
α (deg)	90	90	90	90	90
β (deg)	101.826(7)	109.90(3)	90.857(10)	95.818(7)	101.6521(16)
γ (deg)	90	90	90	90	90
*V* (Å^3^)	874.92(10)	882.4(5)	1567.3(3)	1350.08(17)	1255.96(8)
*D*_*calc*_ (g/cm^3^)	2.255	2.085	2.322	1.954	2.633
μ (mm^–1^)	3.652	3.605	4.059	2.395	5.050
*F*(000)	556	520	1016	764	912
T_max_, T_min_	1.000, 0.765	1.000, 0.620	1.000, 0.839	1.000, 0.895	1.000, 0.844
Θ range for data	2.98–25.35	2.44–25.35	1.68–25.35	2.26–25.34	2.26–30.05
reflections coll.	4363	5215	9018	4583	13 450
data/restr./param.	866/0/74	1617/0/111	2887/0/209	2446/0/190	1841/0/95
*R*(*int*)	0.0189	0.0461	0.0586	0.0289	0.0344
final *R* [*I* > 2σ(*I*)] *R*1, *wR*2	0.0167, 0.0415	0.0388, 0.0842	0.0466, 0.0979	0.0285, 0.0570	0.0175,0.0421
final *R* (all data) *R*1, *wR*2	0.0173, 0.0417	0.0655, 0.1011	0.0759, 0.1232	0.0427, 0.0618	0.0195,0.0427
goodness-of-fit on *F*^2^	1.196	1.077	1.094	1.046	1.294
Δ*ρ*_*max*_, Δ*ρ*_*min*_ (eÅ^–3^)	0.313, −0.589	0.693, −0.776	1.582, −0.901	0.438, −0.433	0.554, −0.501
CCDC Deposition No.	2297606	2297607	2297608	2297609	2297610

**Table 2:** Crystallographic Data for Cocrystals **1B**–**5B**

	**1Bpyz-**O·TIE	**2B2(tmpz-O)·TIE**	**3Bquox-**O·TIE	**4Bphz-**O·TIE	**5Bpyrm-**O·TIE
empirical formula	C_6_H_4_I_4_N_2_O	C_18_H_24_I_4_N_4_O_2_	C_10_H_6_I_4_N_2_O	C_14_H_8_I_4_N_2_O	C_6_H_4_I_4_N_2_O
*M*_*r*_ (g/mol)	627.71	836.01	677.77	727.82	627.71
crystal system	monoclinic	triclinic	triclinic	orthorhombic	orthorhombic
space group, *Z*	*P*2_1_/*c*, 2	*p*-1, 1	*p*-1, 2	*Pna*2_1_, 4	*Pna*2_1_, 4
temperature (K)	170(2)	170(2)	170(2)	170(2)	100(2)
*a* (Å)	12.1172(9)	7.8089(5)	4.3622(2)	30.1132(8)	13.1038(5)
*b* (Å)	4.3378(2)	8.9644(8)	12.4751(4)	4.55913(14)	22.1906(8)
*c* (Å)	12.6539(8)	9.5475(8)	13.8205(6)	12.5319(4)	4.26690(10)
α (deg)	90	81.409(7)	89.793(3)	90	90
β (deg)	110.850(8)	71.016(6)	83.403(4)	90	90
γ (deg)	90	84.741(6)	85.880(3)	90	90
*V* (Å^3^)	621.56(7)	624.24(9)	745.18(5)	1720.50(9)	1240.73(7)
*D*_*calc*_ (g/cm^3^)	3.354	2.224	3.021	2.810	3.360
μ (mm^–1^)	9.995	5.012	8.350	7.244	10.014
*F*(000)	548	388	600	1304	1096
*T*_max_, *T*_min_	1.000, 0.792	1.000, 0.619	1.000, 0.283	1.000, 0.393	1.000, 0.835
Θ range for data	3.27–25.33	2.27–25.34	2.20–25.34	2.11–25.35	2.40–26.43
reflections coll.	4682	5277	6226	13 597	9283
data/restr./param.	1134/43/92	2291/0/141	2731/0/155	3166/1/191	2550/13/120
*R(int*)	0.0374	0.0174	0.0138	0.0261	0.0549
final *R* [*I* > 2σ(*I*)] *R*1, *wR*2	0.0239, 0.0624	0.0356, 0.0876	0.0202, 0.0483	0.0224, 0.0579	0.0284,0.0485
final *R* (all data) *R*1, *wR*2	0.0268, 0.0641	0.0406, 0.0906	0.0221, 0.0491	0.0226, 0.0580	0.0349,0.0505
goodness-of-fit on *F*^2^	1.068	1.108	1.064	1.149	0.988
Δ*ρ*_max_, Δ*ρ*_min_ (eÅ^–3^)	0.688, −1.207	1.811, −1.388	1.435, −1.109	0.645, −0.898	0.943, −0.698
abs. struct. param. (Flack)	-	-	-	0.48(7)	0.18(9)
CCDC deposition no.	2297611	2297612	2297613	2297614	2297615

**Table 3:** Crystallographic Data for Cocrystals **5′A**, **2′B**, and **5′B**

	**5′Apyrm·***p***-F**_**4**_**DIB**	**2′B** tmpz·TIE	**5′B** pyrm·TIE
empirical formula	C_10_H_4_F_4_I_2_N_2_	C_10_H_12_I_4_N_2_	C_6_H_4_I_4_N_2_
*M*_*r*_ (g/mol)	481.95	667.82	611.71
crystal system	monoclinic	monoclinic	orthorhombic
space group, *Z*	*P*2_1_/*n*, 4	*P*2_1_/*c*, 2	*Pbcn*, 4
temperature (K)	100(2)	100(2)	100(2)
*a* (Å)	13.7708(18)	13.7118(8)	4.30020(10)
*b* (Å)	5.7951(8)	4.6574(2)	12.5957(4)
*c* (Å)	16.422(2)	14.0026(8)	21.7443(8)
α (deg)	90	90	90
β (deg)	106.759(4)	118.015(2)	90
γ (deg)	90	90	90
*V* (Å^3^)	1254.8(3)	789.44(7)	1177.76(6)
*D*_calc_ (g/cm^3^)	2.551	2.809	3.450
μ (mm^–1^)	5.044	7.874	10.539
*F*(000)	880	596	1064
*T*_max_, *T*_min_	1.000, 0.747	1.000, 0.764	1.000, 0.791
Θ range for data	2.28–26.04	2.91–30.08	3.23–30.06
reflections coll.	11 468	16 700	15 292
data/restr./param.	2468/0/164	2323/0/75	1703/0/57
*R*(*int*)	0.0437	0.0306	0.0291
final *R* [I > 2σ(*I*)] *R*1, *wR*2	0.0291, 0.0561	0.0141, 0.0282	0.0172, 0.0388
final *R* (all data) *R*1, *wR*2	0.0399, 0.0592	0.0167, 0.0289	0.0181, 0.0390
goodness-of-fit on *F*^2^	1.123	1.113	1.433
Δ*ρ*_max_, Δ*ρ*_min_ (eÅ^–3^)	0.748, −0.609	0.456, −0.508	0.550, −0.604
CCDC deposition no.	2297616	2297617	2297618

### 2.5 Computational Methodology

All computations were performed with the Gaussian 09W suite of programs.^63^ The interaction energies and geometrical parameters were computed using the DFT method with the M062X functional and def2-SVPD basis set for all atoms, which uses effective core potential for elements beyond krypton.^64,65^ All structural minima were confirmed by the absence of imaginary frequencies using vibrational frequency calculations, and Basis Set Superposition Error (BSSE) corrections were performed for all structures in Gaussian 09W.^63^

## 3 Results and Discussion

### 3.1 Structural Descriptions

**Figure 1:**
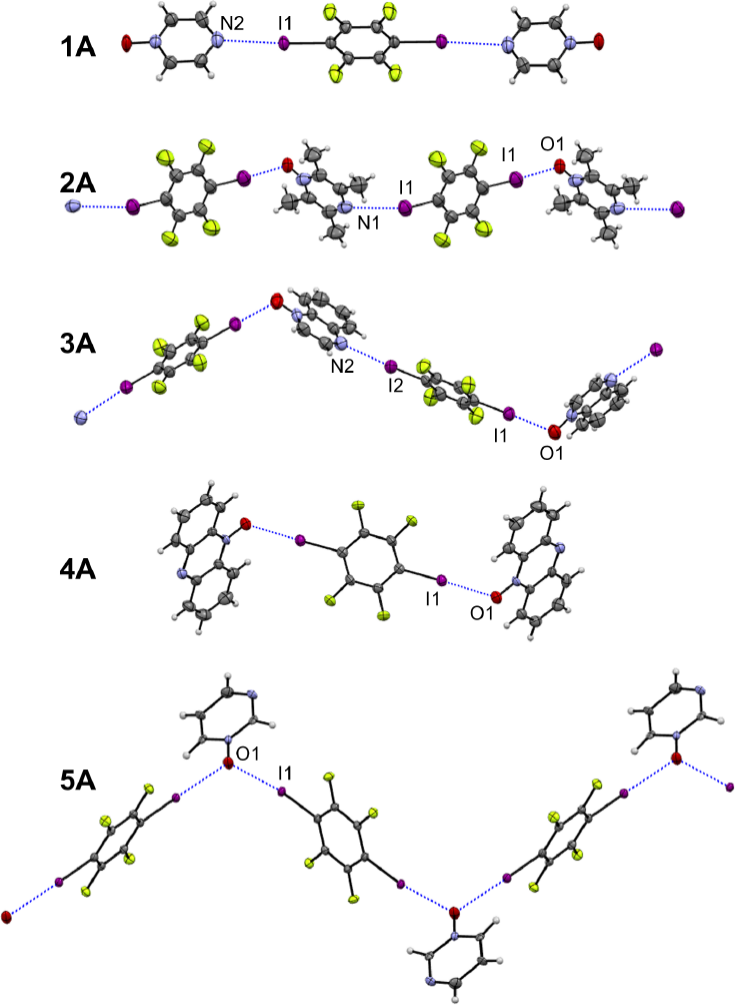
Halogen bonding interactions in cocrystals **1A**–**5A**, with atoms shown as 50% probability ellipsoids.

Halogen bonding interactions for cocrystals **1A**, **2A**, **3A**, **4A**, and **5A** are shown in Figure 1. The asymmetric unit of **1A** consists of half of a mirror-symmetric pyz-O molecule, and half of a mirror- and inversion-symmetric *p*-F_4_DIB molecule. In this way, the compound crystallizes as a 2:1 cocrystal of 2pyz-O·*p*-F_4_DIB, assembling into a discrete 2:1 unit of pyz-O·*p*-F_4_DIB through I···N halogen bonding interactions. The mean plane of the *p*-F_4_DIB molecule in the center of this unit is inclined at 88.56(13)° to the mean plane of the pyz-O molecules flanking it. The oxygen atom of the *N*-oxide participates in C–H···O hydrogen bonding with two neighboring pyz-O molecules. This connects the discrete XB units into a 2D supramolecular sheet of hydrogen and halogen bonding nearly parallel to (4 0 7) (to about 2°) and having a shallow stair-step (0.894 Å) imparted by the C–H···O interactions. Neighboring sheets stack via offset pi···pi interactions of pyz-O molecules at a plane-to-plane distance of 3.383 Å and also via C–H···F interactions. The pyz-O and *p*-F_4_DIB molecules form individual layers in the *ab* plane, where double layers of pyz-O pack in alternating fashion along the *c*-axis with a single layer of *p*-F_4_DIB.

The asymmetric unit of **2A** consists of half of each of a tmpz-O and *p*-F_4_DIB molecule, each having an inversion symmetry. The anisotropic displacement parameters of the oxygen atom indicated it was half-occupied, and thus disordered, to make the tmpz-O molecule compatible with inversion symmetry, and improving the refinement statistics. Chains of tmpz-O and *p*-F_4_DIB molecules propagate along [0 1–1] by alternating I···O and I···N interactions because of the symmetry-imposed disorder. In this way, the I···O (*R*_XB_ = 0.77, where *R*_XB_ is the halogen bond distance normalized to the sum of the van der Waals radii) interactions are considerably shorter than the I···N interactions (*R*_XB_ = 0.92). As they propagate, the chains likewise exhibit alternating deeper and shallower corrugations of about 120° and 138° through the respective I···O and I···N connections. The mean planes of the molecules in the chain are inclined at 67.6(2)° to one another. Neighboring chains maintain C–H···O and C–H···F contacts between one another. In the packing diagram, stacks of tmpz-O and *p*-F_4_DIB molecules alternate along both the *a*- and *c*-axes.

The structure of **3A** is formed through one unique molecule of each of quox-O and *p*-F_4_DIB. Molecules form chains that propagate along the *c*-axis via alternating I···O and I···N interactions. The chains exhibit a corrugation of about 114° that occurs where the I···O halogen bonds propagate the chains and straighten to 167° through the I···N halogen bonds. Neighboring chains maintain short C–H···F contacts as well as offset pi···pi interactions of quox-O molecules having a plane-to-plane distance of 3.314 Å. The quox-O and *p*-F_4_DIB molecules pack in an alternating fashion along the *c*-axis with neighboring rows along the *a*-axis slightly offset from one another.

Cocrystal **4A** is a 2:1 composition of phz-O·*p*-F_4_DIB, with one full molecule of phz-O and one-half molecule of *p*-F_4_DIB in the asymmetric unit. As with the 2:1 cocrystal **1A**, **4A** likewise forms a discrete unit, but in **4A** the halogen bonding occurs with the oxygen atom rather than the nitrogen atom. Hydrogen bonding again complements the halogen bonding interactions, with C–H···N interactions occurring between neighboring units to extend the supramolecular structure into two dimensions. Additional complementary C–H···I and C–H···O interactions connect these sheets. In the packing diagram, stacks of *p*-F_4_DIB molecules occurring along the *a*-axis are fully surrounded by stacks of the phz-O molecules.

The cocrystal of pyrm-O with *p*-F_4_DIB, **5A**, crystallizes in a 1:1 ratio with the pyrm-O molecule sitting on a 2-fold rotation axis and the *p*-F_4_DIB molecule sitting on an inversion center. The pyrm-O molecule was thus disordered by the 2-fold rotation axis about the N–O bond in that the nonoxidized nitrogen atom can occur at either the 3- or 5-position on the pyrimidine ring. One reason for this may be that the halogen bonding motif does not involve the nonoxidized nitrogen atom. The nonoxidized nitrogen atom does not appear to be involved in any short contacts of a complementary nature either. The **5A** cocrystal forms chains of molecules through I···O interactions, where each oxygen atom acts as a halogen bond acceptor for two iodine atoms. This creates a highly corrugated chain propagating along [1 0 1], where the I···O···I angle is 100.84(8)°. Neighboring chains are further connected through complementary C–H···F interactions. Layers of individual pyrm-O and *p*-F_4_DIB molecules in the *ab* plane pack in an alternating fashion along the *c*-axis.

**Figure 2:**
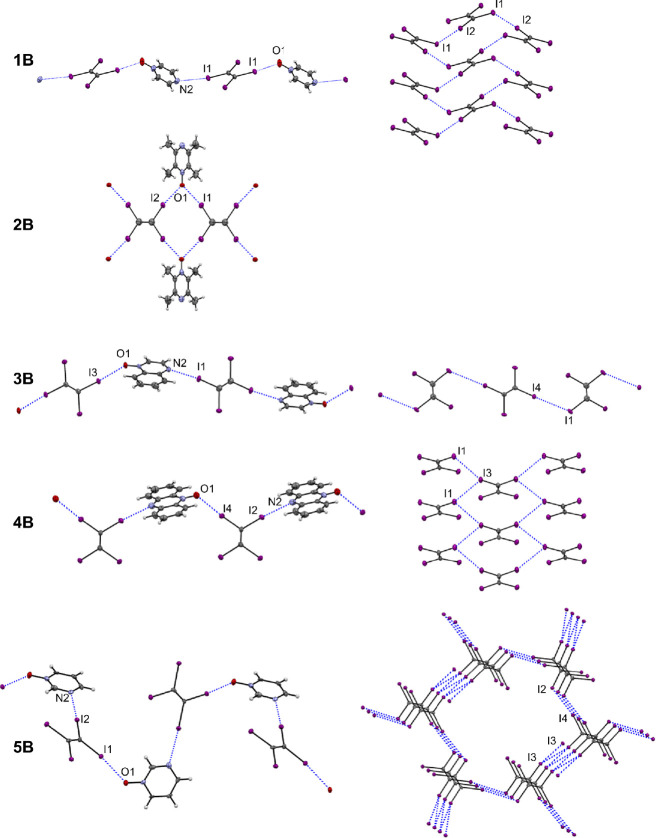
Halogen bonding interactions in the structures of **1B**–**5B**, with atoms shown as 50% probability ellipsoids. Figures in the left column for a given structure show chain formation via I···O and I···N interactions. Figures in the right column show the halogen bonding motifs formed by TIE molecules via I···I interactions.

Halogen bonding interactions in the *N*-oxide cocrystals with TIE are shown in Figure 2. The asymmetric unit of **1B** consists of half of a TIE molecule sitting on an inversion center and a pyz-O molecule that is half-occupied due to disorder over an inversion center. In this way, similar corrugated chains are observed as in **2A**. Chains of TIE and pyz-O molecules propagate parallel to [1 0 1] through alternating I···O and I···N interactions via symmetry-related opposing iodine atoms on the TIE molecule. The additional iodine atoms of the TIE molecule compared to *p*-F_4_DIB provide important I···I intermolecular contacts that form sheets of TIE molecules in the *bc* plane, this has previously been observed with other TIE halogen-bonded cocrystals.^57^ The I1 iodine atoms that act as halogen bond donors toward oxygen and nitrogen atoms of pyr-O serve as halogen bond acceptors for the I2 iodine atoms of neighboring TIE molecules in the TIE sheets. The intersection of the I···O and I···N chains with the I···I sheets creates a three-dimensional halogen-bonded framework. The packing pattern can then be interpreted as sheets of TIE and pyr-O molecules alternating along the *a*-axis.

The asymmetric unit of **2B** consists of one full tmpz-O molecule and one-half of a TIE molecule (completed by inversion symmetry) to create a 2:1 cocrystal stoichiometry of tmpz-O·TIE. The oxygen atom of the tmpz-O molecule exhibits a small degree of disorder over the two nitrogen positions on the pyrazine ring, having a major occupancy attached to N1 of 84% and a minor occupancy attached to N2 of 16%. The major-occupied arrangement is described here. The halogen bonding motif is a one-dimensional chain with TIE molecules at the core of the chain and tmpz-O molecules on the outside. The chains propagate along [1 0 0] via I···O interactions, where each oxygen atom accepts halogen bonds from two iodine atoms of neighboring TIE molecules, occurring at an I···O···I angle of 88.33(14)°. This occurs on both sides of the chain due to the inversion symmetry of the structure, creating the core-clad chain. The I···O halogen bonds thus occupy all of the iodine atoms of the TIE molecule, and no further I···I interactions are observed. The nonoxidized nitrogen atom, N2, does not participate in halogen bonding interactions. In the packing diagram, TIE molecules occupy the corners of the unit cell, while tmpz-O molecules stack in the center of the unit cell.

The cocrystal of quox-O and TIE (**3B)** occurs in a 1:1 stoichiometry of molecules where the quox-O molecule is present in full in the asymmetric unit and TIE is present as two unique half molecules. Chains of alternating quox-O and TIE molecules occur along [0–2 1] via I···O and I···N interactions. These involve the symmetry related, opposing iodine atoms on both unique TIE units (I1 and I3). One of the remaining iodine atoms on one of the TIE molecules, I4, acts as a halogen bond donor, with I1 as its acceptor, to form chains of TIE molecules that intersect with the I···O and I···N chains at the I1 sites where the I···N interactions occur. These TIE chains propagate along [−2 0 1] via the I···I interactions, and exhibit a slight twist as the TIE molecules are inclined at 20.1(2)° to one another. The final unique iodine atom on the TIE molecules, I2, does not participate in any halogen bonding contacts. The intersecting chains form a two-dimensional sheet of I···O, I···N, and I···I halogen bonds parallel to (1 1 2). Molecular packing in **3B** occurs via *ac*-planar slabs of individual TIE and quox-O molecules alternating with one another along the *b*-axis.

The phz-O·TIE cocrystal **4B** consists of one full unique molecule for each of the constituent phz-O and TIE molecules. These molecules form chains propagating along [0 0 1] through alternating I···O and I···N interactions involving two of the iodine atoms of TIE. The TIE and phz-O molecules are inclined at 68.10(5)° to one another. The remaining two iodine atoms of TIE maintain halogen bonding interactions each with two neighboring TIE molecules, as both halogen bond donors and halogen bond acceptors. These I···I interactions form sheets of halogen-bonded TIE molecules parallel to (1 0 0). Since the direction of chain propagation is coincident with one of the dimensions of the TIE sheets, the combination of I···O, I···N, and I···I interactions forms a two-dimensional slab-like motif, with a thickness of half the *a*-axis length. The packing diagram shows phz-O and TIE molecules arranged in an alternating fashion along the *a*-axis, stacked in offset layers along the *c*-axis.

Cocrystal **5B** is a 1:1 stoichiometry of pyrm-O·TIE with one full molecule of each in the asymmetric unit. Chains of molecules are formed along [1 0 2] via I···O and I···N interactions. Unlike **5A**, both the oxygen atom and the nonoxidized nitrogen atom of pyrm-O in **5B** participate in halogen bonding. This imparts a sinusoidal sense to the chains, given the proximity of these halogen bond acceptor sites on the pyrimidine ring. The TIE molecules additionally form their own 3D framework via I···I interactions. The I1 iodine atom that forms the I···O interaction does not participate in I···I interactions, but the I2 iodine atom that forms the I···N acts as a halogen bond acceptor for I4 of a neighboring molecule (and vice versa). The I3 iodine atom forms two I···I interactions with I3 atoms on neighboring TIE molecules: one as a halogen bond donor and one as a halogen bond acceptor. A notable feature of this I···I network is the formation of channels along the *c*-axis that accommodates the pyrm-O molecules, as seen in the packing diagram.

**Figure 3:**
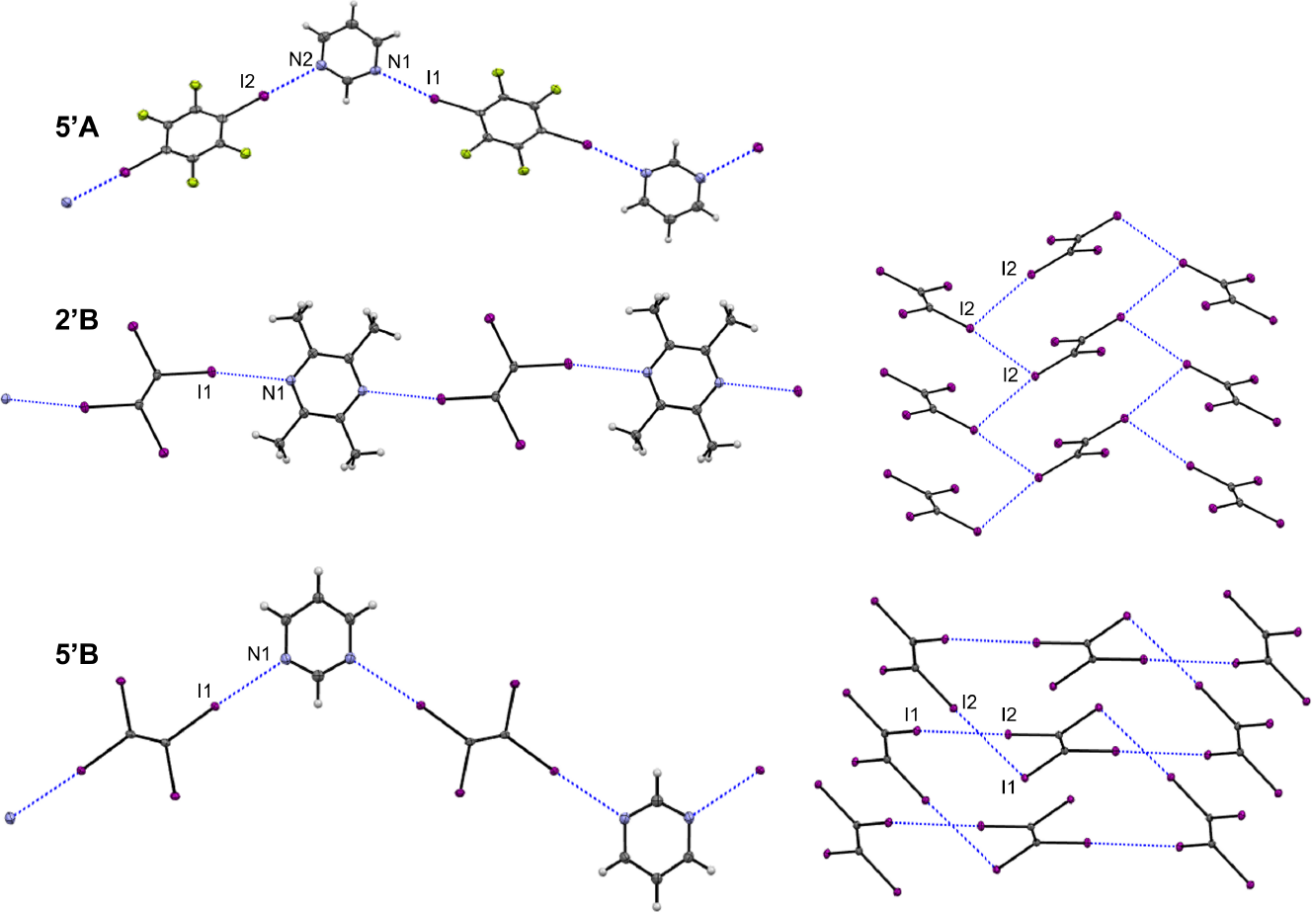
Halogen bonding interactions in the structures of **5′A**, **2′B**, and **5′B**. Figures in the left-hand column for a given structure show chain formation via I···O and I···N interactions. Figures in the right-hand column for **2′B** and **5′B** show the halogen bonding motifs formed by TIE molecules via I···I interactions.

For comparative purposes, cocrystals involving the nonoxidized diazine heterocycles in this study that have not yet been structurally characterized in the literature were also pursued. In this vein, the structures of **5′A**, **2′B**, and **5′B** were also determined, and their halogen bonding interactions are shown in Figure 3. The asymmetric unit of **5′A** consists of one full pyrm molecule and two unique halves of *p*-F_4_DIB molecules. These assemble into chains propagating along [2 0 1] via I···N interactions. Neighboring chains are connected through C–H···F interactions in a three-dimensional fashion. In the packing structure, layers of individual *p*-F_4_DIB and pyrm molecules in the *ab* plane alternate along the *c*-axis.

The structure of **2′B** is constructed from two unique half molecules of tmpz and TIE where the full molecules are generated through inversion symmetry. These molecules form straight chains through I···N interactions of symmetry-related iodine atoms on the TIE molecule. These chains propagate along [1 0 0], with the TIE and tmpz molecules nearly coplanar (inclined at 13.44(14)° to one another). The second unique iodine atom of the TIE molecule interacts with its symmetry equivalents of two neighboring molecules—acting as a halogen bond donor toward one molecule and a halogen bond acceptor from a second molecule. This forms I···I sheets in the *bc* plane. The combination of I···N chains with I···I sheets creates a three-dimensional halogen-bonded framework. This is similar to what was observed in **1B**, except here in **2′B** the tmpz molecules connect the TIE sheets directly along the *a*-axis, rather than in an angled fashion along [1 0 1]. This leads to a thematically similar packing diagram of sheets of TIE and tmpz alternating along the *a*-axis.

The **5′B** cocrystal of pyrimidine and TIE contains half of a unique molecule each of pyrm and TIE in its asymmetric unit. The pyrm molecule is completed by 2-fold rotational symmetry, while the TIE molecule is completed by inversion symmetry. Chains of molecules are formed along [2 0 1] through symmetric I···N interactions, with corrugation imparted by the relative positions of the nitrogen atoms in pyrimidine (compared to straight chains in **2′B**, for example, and similar to the chains in **5′A**). The TIE molecules interact with one another through I···I interactions that form a 2D motif in the *ab* plane. The I2 atom acts as a halogen bond donor toward I1 (which is the halogen bond donor in the I···N interactions) to form these cross-linked sheets. The combination of I···N and I···I interactions thus creates an overall three-dimensional halogen-bonded network. Layers of individual TIE and pyrm molecules in the *ab* plane alternate along the *c*-axis to form a packing structure.

### 3.2 General Halogen Bonding Trends

Several general features are observed over this series of compounds. A summary of the geometric parameters of the halogen bonding interactions is given in Table 4. Most of the cocrystals obtained from these reactions exhibited a 1:1 stoichiometry of *N*-oxide:organoiodine. The exceptions to this were 2:1 cocrystals **1A**, **4A**, and **2B**. For **1A** and **4A** involving the *p*-F_4_DIB organoiodine, we observed the formation of a finite halogen-bonded unit, where *p*-F_4_DIB was sandwiched between the two halogen bond acceptor molecules. We have also observed this tendency elsewhere for 2:1 cocrystal stoichiometries with *p*-F_4_DIB.^66,67^ Interestingly, **1A** and **4A** differ in their formation of I···N versus I···O interactions, and both appear to be fairly strong based on their normalized halogen bond lengths, *R*_XB_ (0.80 and 0.77). The availability of four iodine atoms for halogen bonding in the TIE molecule of **2B** perhaps leads this 2:1 cocrystal to form a different motif than the sandwiched *p*-F_4_DIB molecules, and a core-clad chain formation is instead observed. It is again interesting that this occurs preferentially in favor of I···O interactions. We point out that the nitrogen site of the tmpz-O molecule in **2B** should be sterically accessible, as the 1:1 cocrystal of tmpz with TIE, **2′B**, forms chains of I···N interactions of similar strength to the I···O interactions in **2B** (*R*_XB_ = 0.83 in both cases).

**Table 4:** Geometric Parameters for Halogen Bonding

cocrystal	*d*(I···O) [Å]	*R*_XB_	*d*(I···N) [Å]	*R*_XB_	*d*(I···I) (Å)	*R*_XB_	*∠* (C*–*I···O) (°)	*∠*(I···O*–*N) (°)	*∠* (C*–*I···N) [°]
2(pyz-O)·*p*-F_4_DIB (**1A**)	–	–	2.962(3)	0.80	–	–	–	–	173.91(11)
tmpz-O·*p*-F_4_DIB (**2A**)	2.731(9)	0.77	3.405(8)	0.92	–	–	176.9(3)	114.8(6)	163.3(6)
quox-O·*p*-F_4_DIB (**3A**)	2.841(6)	0.80	2.985(8)	0.81	–	–	170.5(3)	112.7(5)	175.6(3)
2(phz-O)·*p*-F_4_DIB (**4A**)	2.728(3)	0.77	–	–	–	–	178.67(12)	119.1(2)	–
pyrm-O·*p*-F_4_DIB (**5A**)	2.8342(17)	0.80	–	–	–	–	172.15(7)	129.58(4)	–
pyrm·*p*-F_4_DIB (**5′A**)	–	–	2.868(4)2.855(4)	0.78 0.77	–	–	–	–	171.27(16)171.48(17)
pyz-O·TIE (**1B**)	2.793(9)	0.79	3.224(9)	0.87	3.7407(5)	0.92	170.2(2)	122(1)	171.3(2)
2(tmpz-O)·TIE (**2B**)	2.933(5)2.939(5)	0.83 0.83	–	–	–	–	169.6(2)169.4(2)	136.6(4)135.1(4)	–
tmpz·TIE (**2′B**)	–	–	3.0695(15)	0.83	3.7903(2)	0.93	–	–	170.90(6)
quox-O·TIE (**3B**)	2.925(3)	0.83	2.969(4)	0.80	3.9197(4)	0.96	172.30(14)	142.7(2)	171.40(15)
phz-O·TIE (**4B**)	2.925(8)	0.83	3.038(10)	0.82	3.8141(9)3.8562(9)	0.93 0.95	173.4(3)	127.0(7)	174.9(3)
pyrm-O·TIE (**5B**)	2.815(7)	0.80	2.935(9)	0.79	3.6628(10)3.7472(10)	0.90 0.92	171.6(4)	131.6(6)	176.3(3)
pyrm·TIE (**5′B**)	–	–	2.935(3)	0.79	3.7529(3)	0.92	–	–	176.36(10)

A dominant feature of all of the 1:1 stoichiometric cocrystal structures here is the tendency toward one-dimensional I···N and I···O halogen bond motifs. These chains typically propagate via alternating I···N and I···O interactions, excepting cocrystal **5A** where the nitrogen atom is not utilized and the chains propagate instead via I···O···I interactions. Similar to **2B**/**2′B**, pyrimidine shows that it is perfectly capable of forming I···N chains in the structures of **5′A** and **5′B**, and these I···N interactions appear to be some of the strongest I···N interactions found in the current study based on their *R*_XB_. Among the 1:1 cocrystals that form the alternating I···N and I···O chains (**2A**, **3A**, **1B**, **3B**, **4B**, **5B**), we most often observe similar *R*_XB_ values for I···N and I···O, and in fact they have identical median *R*_XB_ values of 0.815. There are two individual exceptions, though, that may point toward a preference toward stronger I···O interactions compared to I···N interactions. The **2A** and **1B** cocrystals both exhibit significantly shorter I···O interactions (*R*_XB_ = 0.77, 0.79) compared to their respective I···N interactions (*R*_XB_ = 0.92, 0.87). Both of these structures are subject to symmetry-imposed disorder of the *N*-oxide oxygen atom, which has the effect of creating on average more space between the molecules to accommodate the disorder. Nevertheless, the tendency of these diazine *N*-oxides to form one-dimensional halogen bonding motifs is similar to what is commonly observed for the nonoxidized diazines.^68^

None of the structures in this study involving *p*-F_4_DIB exhibited any extended I···I interactions. However, a significant number of extended interactions between iodine atoms of neighboring TIE molecules occurred in those cocrystals (**1B**, **3B**, **4B**, **2′B**, **5′B**). Two-dimensional TIE···TIE networks were observed in **1B**, **4B**, **2′B**, and **5′B**, while a one-dimensional motif was found in **3B** and a three-dimensional motif was found in **5B**. All four iodine atoms of TIE participate in some form of halogen bonding interaction in all of the TIE molecules in the TIE-containing cocrystals except one of the two unique TIE molecules in **3B**, where only two of the iodine atoms participate in halogen bonding. The I···I interactions are notably weaker (*R*_XB_ = 0.90–0.96) than the I···N and I···O interactions in this series of structures, as the lone pairs of

electrons on the nitrogen and oxygen acceptor sites appear to strengthen the electrostatic attraction compared with the belt of negative potential on iodine acceptor sites. However, the I···I networks of TIE do appear to be influential in directing the packing nature of these cocrystals, even if the three-dimensional I···I framework of TIE itself is never expressly duplicated in these structures.^57,69−71^ The lattice parameters of TIE (*P*2_1_/*c* polymorph at room temperature, *a* = 15.076(2), *b* = 4.3845(7), *c* = 12.908(1)) are thematic to a certain degree in the cocrystals of **1B**, **3B**, **4B**, **5B**, **2′B**, and **5′B** where TIE···TIE interactions occur in conjunction with two crystallographic axes of ∼4.0–4.7 Å and ∼12.5–14.0 Å.

### 3.3 Computational Analysis of Halogen Bonding

#### 3.3.1 Dinitrogen Heterocyclic Aromatic Mono-N-Oxide Compounds with Organoiodines

Complexes between two organoiodines and five aromatic heterocyclic diazine-mono-*N*-oxides formed via I···N or I···O halogen bonding were optimized using density functional theory (DFT). The optimizations were performed in the gas phases for the various 1:1 *N*-oxide:organoiodine complexes and N:organoiodine complexes at the M062X level of theory using the def2-SVPD basis set. Table 5 lists the energy and selected geometries of the various dinitrogen heterocyclic aromatic mono *N*-oxide complexes that were simulated. The simulation produced structures in which the halogen bond donor and acceptor arrangements were similar to those measured in the crystallographic structures (Table 4 for selected crystallographic geometries). The calculated structures all showed similar arrangements of the donor and acceptor moieties in each class of halogen bonding complex (N–O···I or N···I). In the various simulated complexes, the A···I–C angle (where A = O or N) varied within a narrow range around 180°; the O···I–C angles vary from 179.0° to 171.2°, and the N···I–C angle ranges from 180.0° to 175.9°. For the *N*-oxide complexes, the N–O···I angle ranged from 101.7° to 107.0°, while the C–N···I angle ranged from 114.3° to 128.8°. In these complexes, the interatomic O···I distance varied from 2.803 to 2.865 Å. Halogen bonding interactions resulted in an average increase of 0.017 Å in the *N*-oxide N–O bond. The N···I distance varied from 2.949 to 3.112 Å for the halogen bonding interaction. The C–I distance increased upon halogen bonding for all of the complexes. The average increase for *p*-F_4_DIB (**A**) N–O···I complexes was 0.013 Å and for N···I complexes was 0.014 Å. For tetraiodoethylene (**B**) the increase was 0.009 Å in C–I length for N–O···I complexes and 0.011 Å for N···I complexes. The gas phase interaction energies for the complexes were calculated to range from −26.1 to −32.4 kJ/mol for the N–O···I, and range from −19.2 to −25.4 kJ/mol for N···I. In all complexes, the calculated complex formation energies are lower for the iodine–oxygen halogen bonding complex, with the N–O···I interaction being on average 4.7 kJ/mol lower in energy than the corresponding N···I interaction for a given cocrystal. For all complexes, I···O interactions were calculated to have shorter distances compared to I···N interactions. The average I···O halogen bond distance is 2.84 Å, while the average I···N halogen bond distance is 2.99 Å, a difference of 0.15 Å. This indicates that I···O halogen bonds are generally predicted to be stronger than I···N halogen bonds in these complexes. Simulations suggest that the I···O halogen bonds should be the preferred interaction observed in the X-ray structures, which is indeed the case in **4A**, **5A**, and **2B**, where no I···N interaction was observed. All of the remaining structures except **1A**, form chains of alternating I···O and I···N halogen bonding, optimizing the use of all available interactions. As for **1A**, other packing considerations must be responsible for the choice of I···N over I···O. There is a very weak correlation observed between the energy and bond distance for the I···O halogen bond and the I···N halogen bond in the simulation. This suggests a relatively flat bottom of the energy well for the halogen bonding interaction.

**Table 5:** Halogen Bond Lengths, Angles and Interaction Energies of Cocrystals of Dinitrogen Heterocyclic Aromatic Mono-*N*-oxides with *p*-F_4_DIB and TIE^a^

complex		*d*(I···O)*/d*(I···N) [Å]	*∠(C–*I···O)*/∠(C–*I···N) [deg]	*∠*(I···O*–N*)/*∠*(I···N*–C*) [deg]	*ΔE*(I···O)*/ΔE*(I···N)[kJ/mol]	ref.
2(pyz-O)·*p*-F_4_DIB	**1A**	2.862/2.951	172.2/180.0	102.8/121.8	–26.3/–23.9	this work
tmpz-O·*p*-F_4_DIB	**2A**	2.834/3.112	173.3/180.0	103.6/120.6	–32.3/–24.1	this work
quox-O·*p*-F_4_DIB	**3A**	2.864/2.958	172.7/175.9	102.2/114.3	–27.3/–25.3	this work
2(phz-O)·*p*-F_4_DIB	**4A**	2.834/2.998	171.8/179.9	104.0/121.4	–27.7/–25.2	this work
pyrm-O·*p*-F_4_DIB	**5A**	2.834/2.988	171.6/177.4	105.9/124.0	–30.5/–20.8	this work
pyz-O·TIE	**1B**	2.865/2.957	171.2/178.1	101.7/121.2	–26.1/–22.0	this work
2(tmpz-O)·TIE	**2B**	2.830/3.044	173.2/178.6	103.6/117.4	–31.9/–24.2	this work
quox-O·TIE	**3B**	2.851/2.949	172.8/177.7	103.5/128.8	–26.1/–25.0	this work
phz-O·TIE	**4B**	2.803/2.988	175.9/179.0	107.0/121.3	–26.4/–24.2	this work
pyrm-O·TIE	**5B**	2.826/2.997	171.5/176.5	102.3/115.8	–29.4/–19.2	this work

a Optimized at the M062X/def2-SVPD level of theory.

#### 3.3.2 Dinitrogen Heterocyclic Aromatic Compounds with Organoiodines

To further understand the context of the N–O···I interactions versus N···I interactions, additional simulations were performed for complexes between the organoiodines and the same five dinitrogen aromatic heterocycles without the *N*-oxide functional group. The complexes formed via I···N halogen bonding were optimized by using density functional theory (DFT). The optimizations were performed in the gas phase for the various 1:1 heterocycle:organoiodine complexes at the M062X level of theory using the def2-SVPD basis set. Table 6 lists the energy and selected geometries of the various dinitrogen heterocyclic aromatic complexes that were simulated. The calculated structures all showed similar arrangements of the donor and acceptor moieties in the N···I halogen bonding complexes. In the various simulated complexes, the N···I–C angle varied from 180.0 to 175.3,°, while the C–N···I angle ranged from 114.4° to 122.3°. The interatomic N···I distances varied from 2.942 to 3.096 Å, with an average increase in the C–I bond distance of 0.016 Å for *p*-F_4_DIB N···I complexes and 0.013 Å for TIE N···I complexes. The gas phase interaction energies of the complexes are calculated to range between −21.8 and −25.8 kJ/mol for the N···I. Compared to the *N*-oxide complexes, the nonoxidized complexes exhibit an average reduction in complexation energy by 0.75 kJ/mol and feature a halogen bond distance that is shorter by 0.003 Å.

**Table 6:** Halogen Bond Lengths, Angles and Interaction Energies of Cocrystals of Dinitrogen Aromatic Heterocycles with *p*-F_4_DIB and TIE^a^

complex		*d*(I···N) [Å]	*∠(C–*I···N) [deg]	*∠*(I···N*–*C) [deg]	*ΔE*(I···N)[kJ/mol]	ref.
pyz·*p*-F_4_DIB	**1′A**	2.942	180.0	121.2	–24.7	(53)
tmpz·*p*-F_4_DIB	**2′A**	3.096	179.9	120.5	–24.8	(54)
quox·*p*-F_4_DIB	**3′A**	2.964	176.3	114.4	–21.8	(53)
phz·*p*-F_4_DIB	**4′A**	3.044	180.0	121.2	–24.7	(55)
pyrm·*p*-F_4_DIB	**5′A**	2.948	179.1	122.3	–25.0	this work
pyz·TIE	**1′B**	2.951	175.3	120.6	–23.2	(56)
tmpz·TIE	**2′B**	3.006	178.4	117.0	–25.8	this work
quox·TIE	**3′B**	2.954	175.9	112.7	–25.6	(57)
phz·TIE	**4′B**	3.054	178.4	120.9	–22.6	(57)
pyrm·TIE	**5′B**	2.949	177.4	120.3	–23.3	this work

^a^ Optimized at the M062X/def2-SVPD level of theory.

**Figure 4:**
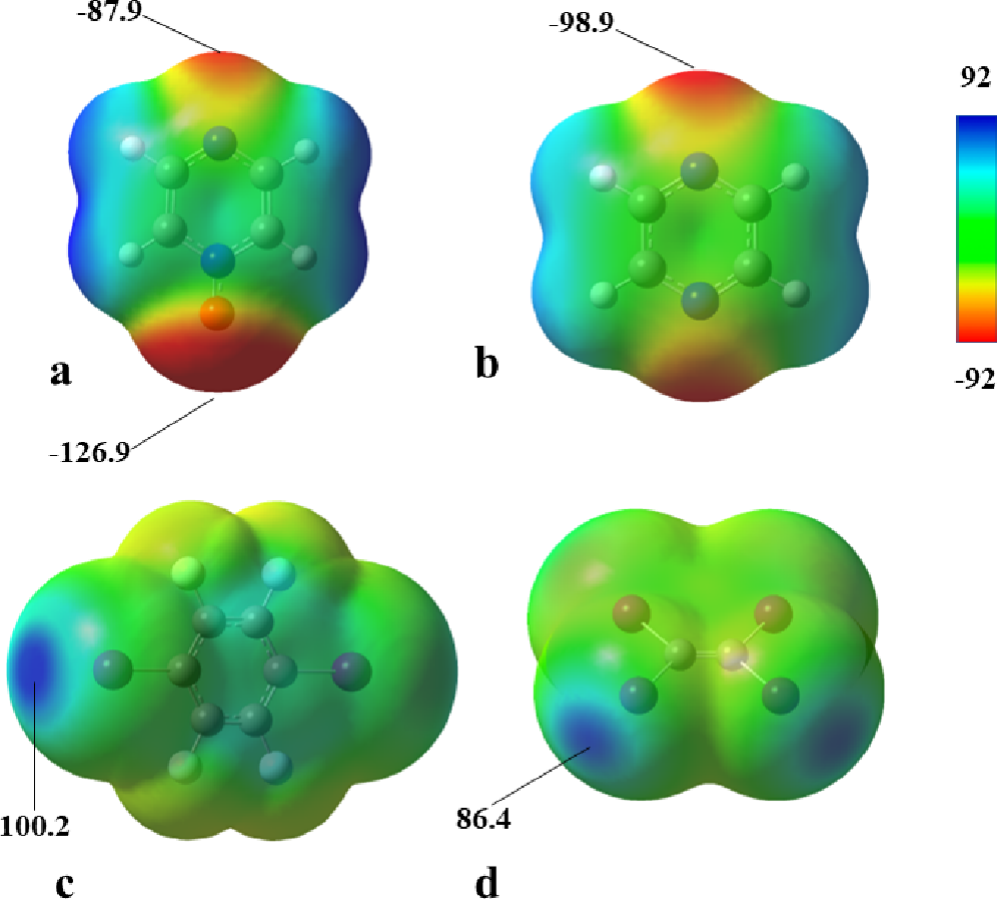
Electrostatic potentials projected on the total electron density surface for geometry optimized a) **1**, b) **1′**, c) **A**, and d) **B**. All images are colored using the scale for *V*, and units are kJ/mol. All calculated at the M062X/def2-SVPD level of theory (isosurface 0.004 au).

**Figure 5:**
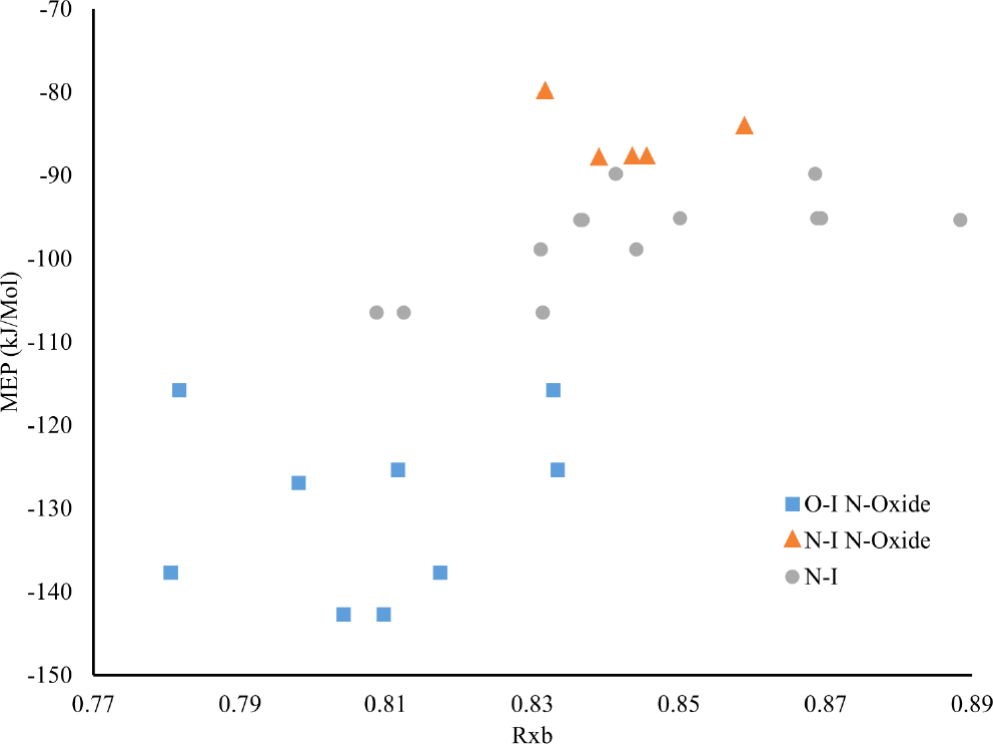
Graphical representation of Rxb compared to halogen bond acceptor molecular electrostatic potential (MEP). Data are color-coded by the halogen bond type (N···I (orange triangles) vs O···I (blue squares) in aromatic *N*-oxide complexes and N···I (gray circles) in dinitrogen heterocycle complexes).

**Figure 6:**
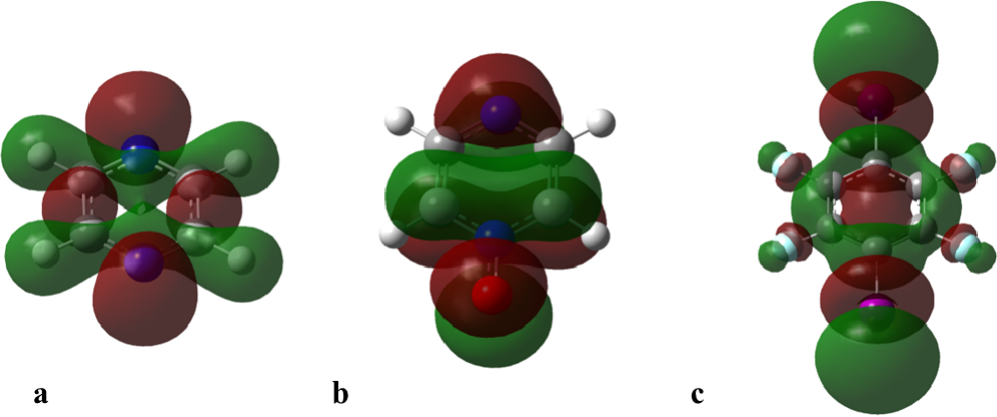
Frontier (HOMO/LUMO) orbital shapes of (a) HOMO **1′**, (b) HOMO **1**, and (c) LUMO **A.** All calculated at the M062X/def2-SVPD level of theory (isosurface 0.004 au).

To investigate what factor influences the O···I and N···I halogen bond strengths, electrostatic potential surfaces were generated (Figure 4) for pyz-O (**1**) and pyz (**1′**). The largest negative potential for **1** was located above the *N*-oxide oxygen atom along the extension of the N–O bond and had a value of *V*_min_ = −126.9 kJ mol^–1^ while the nitrogen atom opposite of the ring from the *N*-oxide group had a *V*_min_ = −87.9 kJ mol^–1^. For comparison, the *V*_min_ of **1′** is located above the nitrogen atoms and has a value of −98.8 kJ mol^–1^. The two donors used in this study have *V*_max_ located above the iodine atoms along the extension of the C–I bond with *V*_max_ = 100.2 kJ mol^–1^ for **A** and *V*_max_ = 86.4 kJ mol^–1^ for **B**. Others have shown that there is a weak correlation between *V*_min_ and the halogen bond interaction energy or the O···I for *N*-oxide I_2_ halogen bonds,^47^*N*-oxide TIE halogen bonds,^48^ and halogen bonds in general.^2^ We observe that trend as well, as shown in Table 7 and Figure 5. By inspection of the electrostatic potentials, one could surmise that the ideal N–O···I and the C–I···O should both be approximately 180°, and the C–N···I and C–I···O angles would be 120° and 180°, respectively, as suggested by Figure 4. However, electrostatics are not the only consideration; the geometries of the complexes are governed by the interaction of the molecular orbitals. In particular the HOMO of the halogen bonding donor and the LUMO of the halogen bonding acceptor guide the geometries of the halogen-bonded complex. In Figure 6, the HOMO of **1′** (a) and **1** (b) are shown, as well as the LUMO of **A** (c). The position of the orbitals suggests the **1′A** complex would have a C–N···I angle of 120° whereas **1A** would have a N–O···I angle of 90°.

**Table 7:** Maximum and Minimum Electrostatic Potential for the Halogen Bond Donors and Acceptors Used in this Study

molecule		*V*_min(O)_ (kJ/mol)	*V*_min(N)_ (kJ/mol)	*V*_max(I)_ (kJ/mol)
pyz-O	**1**	–126.9	–87.9	
tmpz-O	**2**	–137.7	–82.5	
quox-O	**3**	–125.3	–87.8	
phz-O	**4**	–115.8	–84.2	
pyrm-O	**5**	–142.7	–80.0	
pyz	**1′**		–98.9	
tmpz	**2′**		–95.1	
quox	**3′**		–95.3	
phz	**4′**		–89.8	
pyrm	**5′**		–106.5	
*p*-F_4_DIB	**A**			100.2
TIE	**B**			86.4

## 4 Conclusions

The structures of ten new cocrystals of heterocyclic diazine mono-*N*-oxides with the organoiodines *p*-F_4_DIB and TIE were reported. Additionally, the structures of three new cocrystals of heterocyclic diazines with *p*-F_4_DIB and TIE that were not previously reported in the literature were reported here for comparative purposes. All of the structures feature halogen bonding as the key intermolecular interaction between molecules. Most often, though not exclusively, one-dimensional motifs involving both I···O and I···N interactions are observed. In the case of the TIE cocrystals, these one-dimensional features are extended into higher dimensionality through additional I···I interactions.

The experimental data indicate the I···O and I···N interactions are of similar strength and generally stronger than any supporting I···I interactions. X-ray analysis showed that of the aromatic heterocyclic diazine mono-*N*-oxides organoiodine cocrystals examined, six exhibited 1:1 stoichiometry (**2A, 3A, 1B, 3B, 4B, 5B)**, forming chains that utilized both N···I and O···I interactions. Two cocrystals presented a 1:1 stoichiometry with exclusive O···I interactions (**5A, 2B)**. Two cocrystals displayed a 2:1 stoichiometry—one characterized solely by O···I interactions (**4A)** and the other solely by N···I interactions (**1A)**.

Computational studies indicate an energetic preference for I···O interactions over I···N interactions in the optimized structures. These simulations yielded gas-phase structures with geometries that align with our crystallographic findings. The electrostatic potential surfaces show a weak correlation between *V*_min_ and the halogen bond interaction energy, consistent with the correlation that others have observed between the electrostatic potential and the strength of the halogen bonds, also indicating an energetic preference for I···O interactions over I···N interactions. In addition, a computational analysis of the complexes gave formation energies that were, on average, 4.7 kJ/mol lower for the I···O halogen bonding interaction as compared to the corresponding N···I interaction, which also resulted in a decrease of 0.15 Å on average for the I···O interaction distances compared to the I···N interaction distances.

In conclusion, this investigation provides insight into the relative strength of the I···O and I···N halogen bonding interactions in diazine *N*-oxide cocrystals. The interactions are of similar strength, with I···O predicted to be slightly stronger. However, other factors, such as steric and packing considerations, can influence the selection of the halogen bonds observed in the cocrystals. The I···O halogen bonds of *N*-oxides prove to be an important tool in the systematic design of crystal structures and crystal engineering.

## Supporting Information Available

Crystal packing diagrams of the novel structures presented above (PDF)
