# Supplementary material for: Comparison of N···I and N···O Halogen Bonds in Organoiodine Cocrystals of Heterocyclic Aromatic Diazine Mono-N-oxides
Source: Cryst Growth Des. 2024 Mar 5;24(6):2425–38. doi: 10.1021/acs.cgd.3c01344 (PMC10958445; doi:10.1021/acs.cgd.3c01344)
Supplement: Supplementary file 1 — cg3c01344_si_001.pdf [file cg3c01344_si_001.pdf]

Supporting Information

Comparison of N···I and N···O halogen bonds in organoiodine cocrystals of heterocyclic aromatic diazine mono-*N*-oxides.

Clifford W. Padgett,*,‡ Riley Dean,† Audrey Cobb,† Aubree Miller,† Andrew Goetz,‡ Sam Bailey,‡ Kyle Hillis,‡ Colin McMillen,† Sydney Toney,‡ Gary L. Guillet,‡ Will Lynch,‡ William T. Pennington†

†Department of Chemistry, Clemson University, Clemson, SC 29634-0973, USA

‡ Department of Biochemistry, Chemistry and Physics, Georgia Southern University, Savannah GA 31419, USA

Corresponding author email cpadgett@georgiasouthern.edu

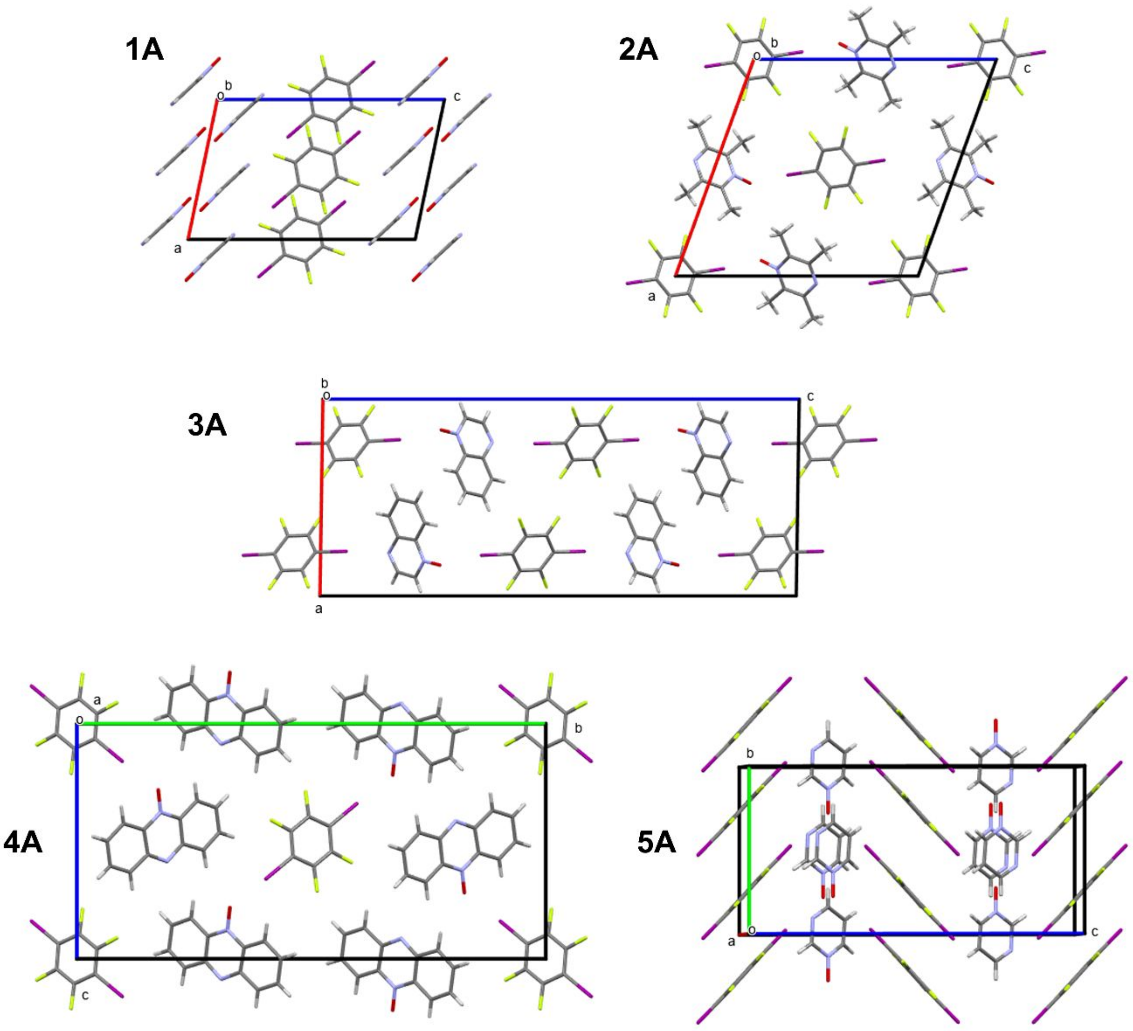

Figure S1. Packing diagrams for cocrystals **1A**-**5A**.

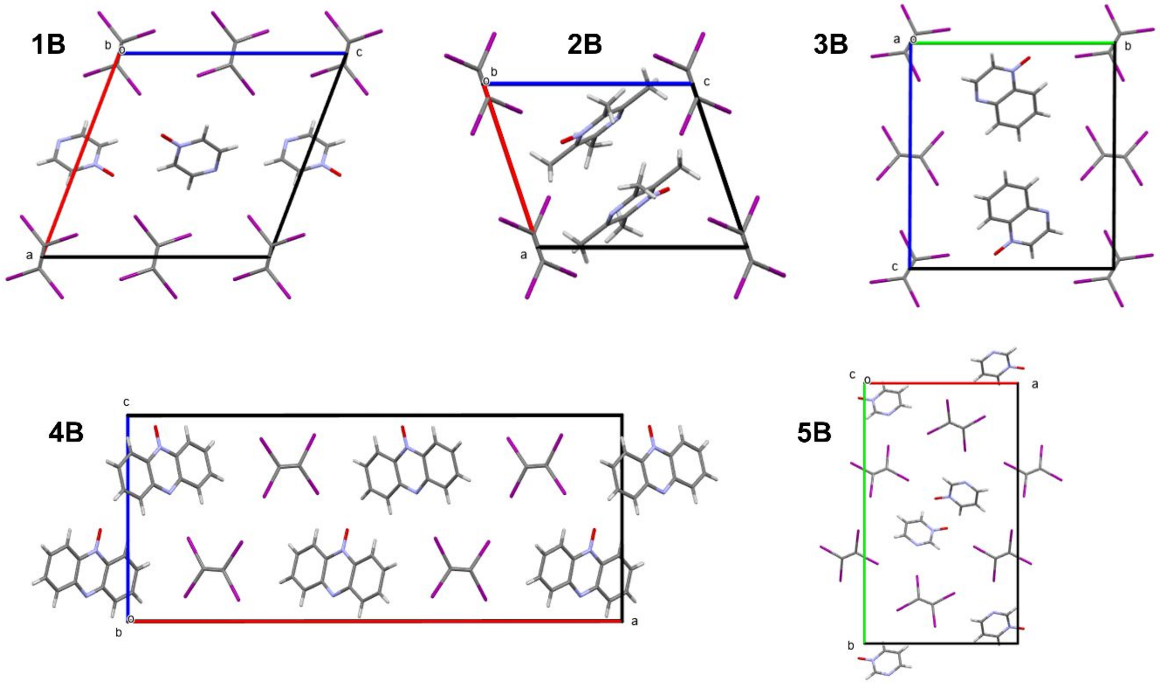

Figure S2. Packing diagrams for cocrystals **1B-5B**.

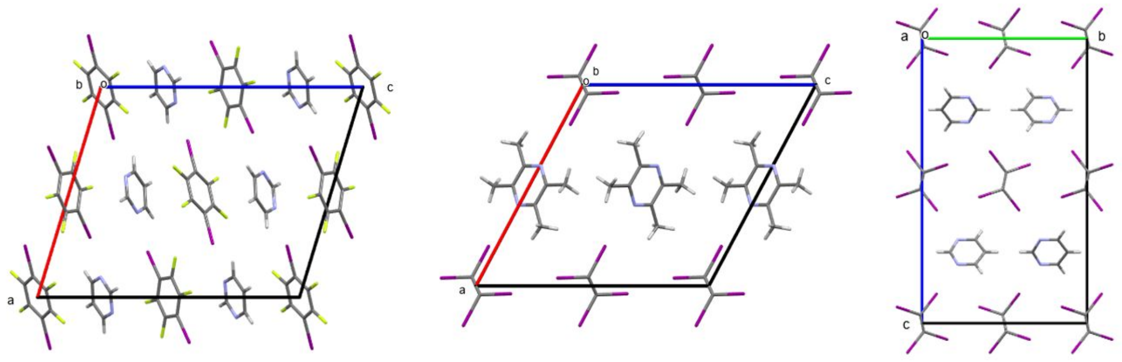

5’A **2’B** **5’B**

Figure S3. Packing diagrams for cocrystals **5’A**, **2’B**, and **5’B**.
